# Two-Photon Voltmeter for Measuring a Molecular Electric Field**

**DOI:** 10.1002/anie.201502157

**Published:** 2015-05-08

**Authors:** Aleksander Rebane, Geoffrey Wicks, Mikhail Drobizhev, Thomas Cooper, Aleksander Trummal, Merle Uudsemaa

**Affiliations:** Deptartment of Physics, Montana State University264 EPS, Bozeman, MT 59717 (USA); National Institute of Chemical Physics and BiophysicsTallinn (Estonia); Air Force Research Lab, Wright Patterson Air Force BaseDayton, OH (USA); Tallinn Institute of TechnologyTallinn (Estonia)

**Keywords:** intramolecular charge transfer, molecular reaction field, solvatochromism, solvent effects, two-photon absorption spectroscopy

## Abstract

We present a new approach for determining the strength of the dipolar solute-induced reaction field, along with the ground- and excited-state electrostatic dipole moments and polarizability of a solvated chromophore, using exclusively one-photon and two-photon absorption measurements. We verify the approach on two benchmark chromophores *N*,*N*-dimethyl-6-propionyl-2-naphthylamine (prodan) and coumarin 153 (C153) in a series of toluene/dimethyl sulfoxide (DMSO) mixtures and find that the experimental values show good quantitative agreement with literature and our quantum-chemical calculations. Our results indicate that the reaction field varies in a surprisingly broad range, 0–10^7^ V cm^−1^, and that at close proximity, on the order of the chromophore radius, the effective dielectric constant of the solute–solvent system displays a unique functional dependence on the bulk dielectric constant, offering new insight into the close-range molecular interaction.

The electronic properties of polar chromophores in solution are dominated by their interactions with the solvent environment. Understanding how the molecular electric dipole moment and polarizability behave in the ground and excited states is critical for optimizing solvent-dependent properties of materials, including absorption and fluorescence spectra as well as photo-initiated charge separation.[1] Furthermore, if the molecular dipoles and polarizabilities in the ground- and excited states could be accurately determined, then spectroscopy would directly probe the intrinsic electric fields acting on the chromophore,[2] thus providing important quantitative insight into solvation, catalysis, and other phenomena.

Among available experimental methods, standard solvatochromism relies on several assumptions, which are typically valid only for a limited range of rigid systems.[1] Electrochromism and microwave conductivity measurements require strong external electric fields and suffer from reduced fidelity in heterogeneous environments.[3] Spectral hole-burning Stark spectroscopy[2c] offers higher selectivity and fidelity, but requires cooling of the samples to cryogenic temperatures to achieve narrow homogeneous line shapes, thus limiting its versatility, especially regarding biological systems. The vibrational Stark effect and vibrational absorption spectroscopy has been used to probe local electric fields, provided that the vibrational frequency shifts are calibrated with respect to external fields.[4] In addition, currently available techniques suffer from ambiguity regarding the boundary separating the core chromophore from its immediate surrounding solvent thus complicating distinction between the intrinsic electric field versus the externally applied voltage.[2a]

Here we report an all-optical method that determines the strength of the dielectric reaction field (*E*_reac_), the vacuum molecular electric dipole moment (*μ*_vac_) and polarizability (*α*) in both the ground- (*S*_0_) and lowest excited (*S*_1_) singlet states, and provides quantitative estimate of the effective molecular size (*a*_0_) for two benchmark polar chromophores, prodan and C153. Our approach combines solvatochromic one-photon absorption (1PA) and femtosecond two-photon absorption (2PA) experiments, where the latter serves as a versatile alternative to the Stark effect and related techniques.[5]

Details of the spectroscopic procedures, spectral data analysis, and computational methods are given in the Supporting Information. Figure 1 shows the 1PA and 2PA spectra of prodan (left) and C153 (right) in three representative mixtures of toluene and DMSO. Both prodan and C153 show a systematic red-shift and broadening of the absorption band with increasing *ε*. Gaussian decomposition of the 1PA spectrum yields the dependence of the peak frequency and the bandwidth of the lowest energy component (dashed-dotted line) on *ε*. By extrapolating these dependencies to *ε*=1 we determined the vacuum peak transition frequency (*ν*_vac_) and the effective “vacuum” spectral bandwidth (Δ*ν*_vac_; Table 1). Here and in the following, we refer to spectral parameters or band shape of the lowest-energy transition band only.

**Figure 1:**
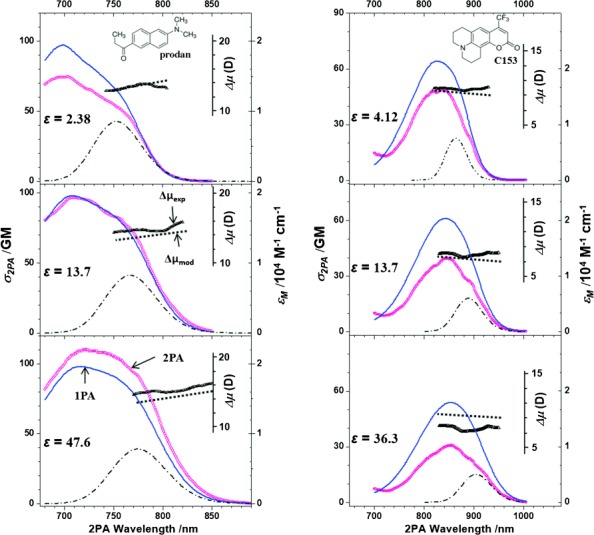
1PA spectrum (blue line) and 2PA spectrum (pink symbols) in prodan (left) and C153 (right), each in three representative toluene:DMSO mixtures. Gaussian fit to the lowest energy band of the *S*_0_→*S*_1_ transition (dash-dotted line); measured Δ*μ* dependence (black symbols, inserted vertical scale); predicted Δ*μ* dependence (thick dotted line).

**Table 1:** Comparison of experimental, calculated, and literature parameter values. Literature computational values are given in italics.

	Prodan			C153		
parameter	exp.	calc.	lit.	exp.	calc.	lit.
*ν*_vac_ [cm^−1^]	26 860±200	31 759[a]		23 505±200	29 344[a]	
*Δν*_vac_ [cm^−1^]	1190±15			440±10		
α(*S*_0_) [Å^3^]	27±3	29.8[b]	27.5[9]	27±3	29.9[b] 30.7[c]	29.7[9]
Δ*α* [Å^3^]	46.8±4.7	36.4[a] (vacuum) 40.2[a] (Toluene)		−6.6±0.7	5.0[a]	5.3±14^[10]^ *4.4*[11]
*μ*_vac_ (*S*_0_) [D]	5.8±0.6	6.13[a]	5.2[3a]	6.7±0.7	7.21[b] 7.27[c]	6.55^[12]^ *6.97*[11]
Δ*μ*_vac_ [D]	12.8±0.6	4.2[a] (vacuum) 6.0[a] (Toluene)	4.4–5.0^[3a]^ 8^[13]^ 12.73[3b]	9.4±0.5	5.4[a]	4.9^[14]^ 6.0–9.5^[10, 15]^ *4.88*^[11]^ *7.9*[16]
*a*_0_ [Å]	6.5±0.7	6.3[b]		4.7±0.5	4.9[b]	*4.41*^[11]^ 4.6[17]
Δ*a* [Å]	1.8			0.08		
*p*	0.65			0.8		

[a] CAM-B3LYP/6-311++G(d,p)//B3LYP/6-311G(d,p).[8]

[b] B3LYP/6-311G(d,p)//B3LYP/6-311G(d,p).[8]

[c] CAM-B3LYP/6-311++G(d,p)//CAM-B3LYP/6-311G++(d,p)[8] (see the Supporting Information).

A two-level model has been shown to provide quantitative description of 2PA in the lowest-energy (0-0) component of S_0_→S_1_ transition of polar chromophores (see the Supporting Information for details), where the 2PA cross section, *σ*_2PA_(2*λ*), is related to the permanent dipole moment change, Δ*μ=μ*(*S*_1_)−*μ*(*S*_0_) (in Debye)[6] given in Equation (1),

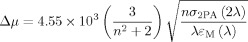
(1)

where *n* is the solvent index of refraction, *λ* is the transition wavelength (nm), *ε*_M_ is the molar extinction coefficient (m^−1^ cm^−1^), and σ_2PA_ is the 2PA cross section, expressed in Göppert–Mayer units (1 GM=10^−50^ cm^4^ photon^−1^ s^−1^). We assume here and in the following that all vector and tensor quantities are aligned with their predominant component pointing along the same coordinate axis. The black symbols in Figure 1 (Δ*μ*_exp_) show the value of Δ*μ* calculated from Equation (1) (inserted vertical axis). In the case of prodan, Δ*μ* shows up to a 15 % increase with increasing wavelength, whereas in C153 the value remains approximately constant.

The solute static dipole polarizes the surrounding dielectric (solvent), which in turn creates a reaction electric field that shifts the energy levels of the chromophore via Stark effect, which then relates to the observed transition frequency change as given in Equations (2) and (3),

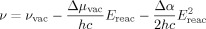
(2)

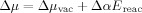
(3)

where *h* is Planck’s constant, *c* is the velocity of light in vacuum, Δ*μ*_vac_ is the vacuum dipole moment change, Δ*α*=α(*S*_1_)−α(*S*_0_) is the change of polarizability, and *E*_reac_ is the solvent field created by *μ*(*S*_0_).

Figure 2 shows the experimental correlation between the peak transition frequency and the average Δ*μ*. Fitting with Equations (2) and (3) gives the values of Δ*μ*_vac_ and Δ*α* (Table 1).

**Figure 2:**
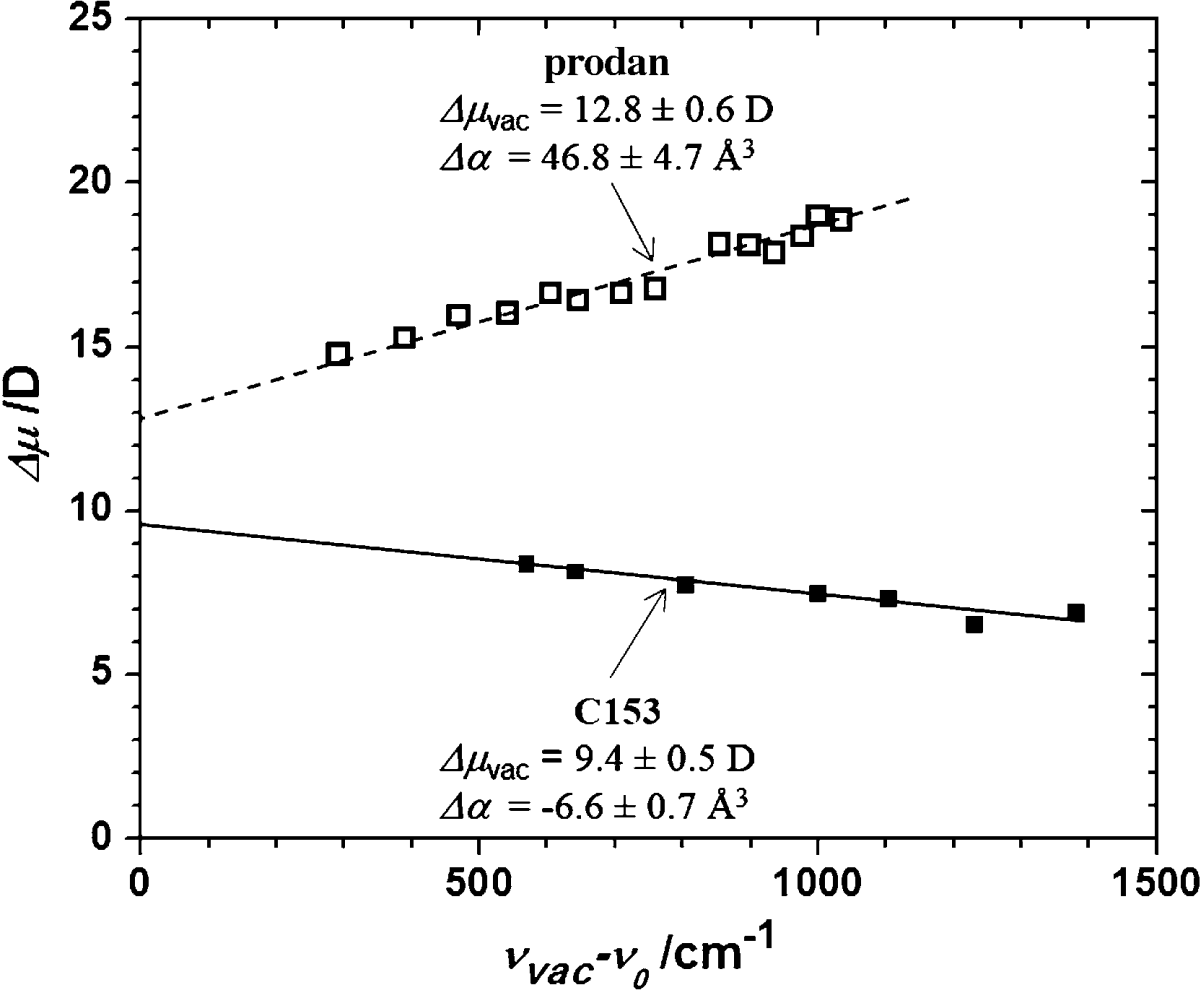
Dependence of Δ*μ* in prodan (empty squares) and C135 (full squares) on *ν*_vac_−*ν*_0_. Extrapolation of the fits to *ν*_vac_ yields the vacuum dipole moment changes, Δ*μ*_vac_=12.8±0.6 D for prodan (dashed line) and Δ*μ*_vac_=9.4±0.5 D for C153 (solid line). The slopes of the fits then give the values for change in polarizability, Δ*α*=46.8±4.7 Å^3^ (prodan) and Δ*α*=−6.6±0.7 Å^3^ (C153).

In order to obtain the vacuum ground-state dipole moment, *μ*_vac_(*S*_0_), and the ground-state polarizability, α(*S*_0_), we invoked a simple phenomenological model that treats the chromophore as a polarizable point dipole embedded in a dielectric continuum in the center of a spherical volume of radius *a*. The strength of the reaction field acting on the dipole is given as Equation (4)]:[7]

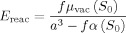
(4)

where *f* represents the dependence on the dielectric constants outside and inside the volume. To account for unknown close-range solute–solvent interaction, while still complying with Maxwell’s equations, this function may be written in the form of Equation (5)]

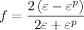
(5)

and where the power dependence (0≤*p*≤*1*) ensures that the direction of the reaction field energetically stabilizes the system while satisfying the vacuum limit [Eq. (6)].

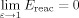
(6)

According to Equations (2) and (4), the transition frequency varies as given in Equation (7).

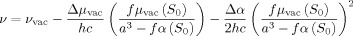
(7)

Solvated chromophores experience heterogeneous and fluctuating solvent environments. We can quantify the local environments by a distribution of effective cavity size, *P*(*a*), which is related to the distribution of transition frequencies, *P*(*ν*), as shown in Equation (8).

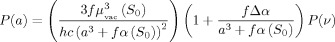
(8)

The transition frequencies are assumed to follow a Gaussian distribution [see Eq. (9)],

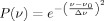
(9)

where *ν*_0_ and Δ*ν* are the center frequency and the half-width of the transition band, respectively. Combining Equations (7)–(9), and defining *E*_0_*=E*_reac_(*a*_0_) and *E*_Δ_=*E*_reac_(*a*_0_+Δ*a*) as the reaction field values that correspond to the peak (*ν*_0_) and half-width (*ν*_0_+Δ*ν*) of the distribution, respectively, we arrive at the distribution of the cavity size *a* [Eq. (10)],

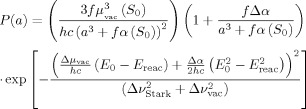
(10)

where Δ*ν*_Stark_ is defined by Equation (11).

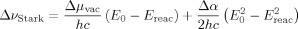
(11)

Inserting our experimental values Δ*μ*_vac_, Δ*α*, *ν*_vac_, and Δ*ν*_vac_ into the model and performing global fitting of the calculated 1PA profiles to the experimental shapes (see the Supporting Information for details), we obtain the remaining parameters, *μ*_vac_(*S*_0_), *α*(*S*_0_), *a*_0_, Δ*a*, and *p* (Table 1). The experimental ground-state values *μ*_vac_(*S*_0_) and *α*(*S*_0_) agree quantitatively with the corresponding calculated values and the literature data, both for prodan and C153. The experimental Δ*μ*_vac_=12.8±0.6 D for prodan and Δ*μ*_vac_=9.4±0.5 D for C153 agree well with previous measurements, however, are about a factor of two above our calculated values. In prodan the experimental polarizability change, Δ*α*, agrees very well with the calculated value, whereas in C153 the value is much smaller, making it effectively close to zero. Despite the fact that we made no a priori assumptions about the size of the molecule, our estimated average cavity size, *a_0_*, agrees surprisingly well with the calculated molecular dimensions. For prodan, we calculated a molecular radius of 6.3 Å,[8] which compares well to our experimental value, *a*_0_=6.5±0.7 Å. Similarly, for C153 we calculated the effective radius in the direction F=C⋅⋅⋅N=C=H to be 4.9 Å, which is also very close to our experimental value, *a*_0_=4.7±0.5 Å.

As a further independent check, we used Equations (2) and (3) to predict how Δ*μ* changes as a function of *ν* within the absorption band (thick dotted line in Figure 1). The results are in good agreement with the measured dependence, and demonstrate that Δ*μ* is not necessarily constant, but may vary within the band.

Figure 3 shows the distribution of *E*_reac_, which appears to vary in a broad range, 0–10^7^ V cm^−1^, essentially caused by diverse local environments. Fluctuations of dielectric environment were previously used to explain temporal fluctuations of the fluorescence lifetime in single molecules.[18] Both prodan and C153 show increase of average *E*_reac_ with increasing *ε*, with the respective maximum values, *E*_reac_≈5.0×10^6^ and 1.0×10^7^ V cm^−1^. The larger reaction field in C153 is due to the larger ground-state dipole moment as well as because of smaller *a*_0_. Also, C153 lacks the flexibility of prodan’s propyl- and dimethylamino groups, which is reflected in its smaller Δ*a* and narrower *E*_reac_ distribution.

**Figure 3:**
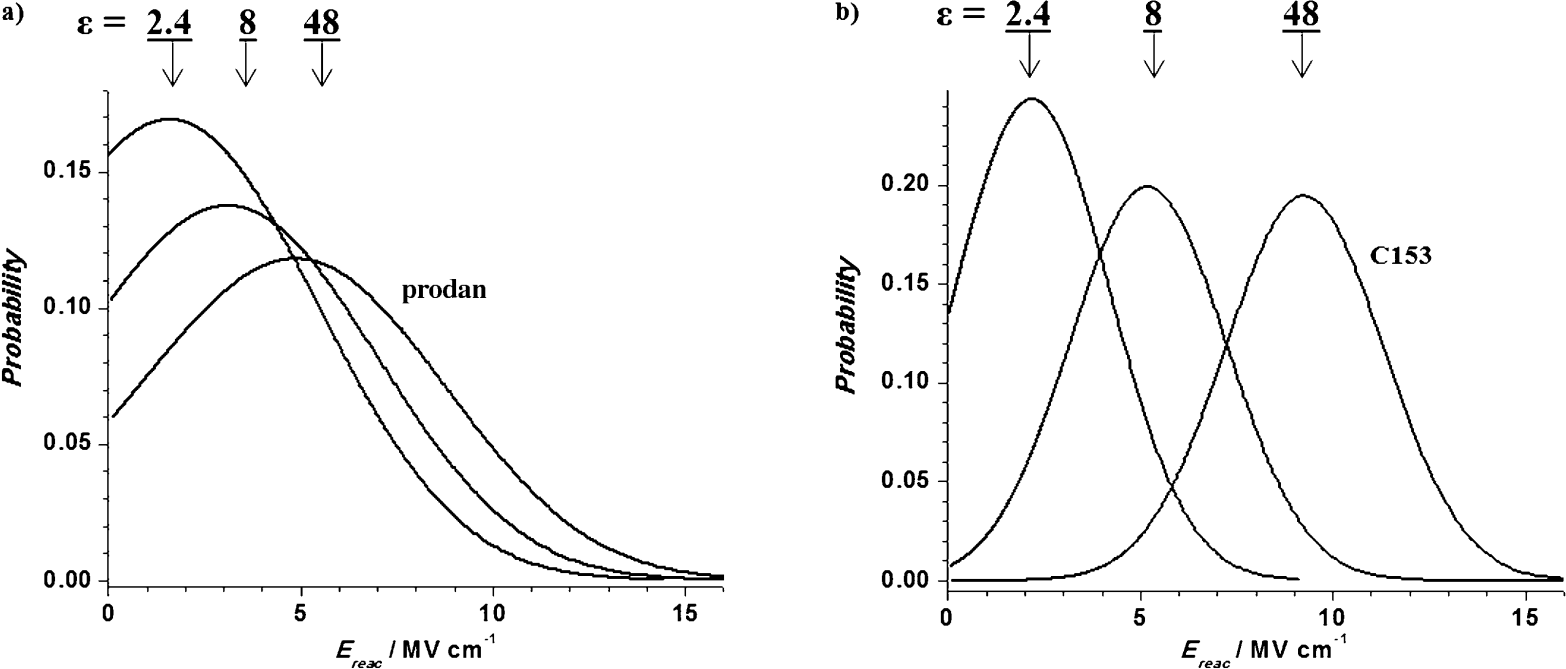
Probability density of the reaction field for different *ε* for a) prodan and b) C153.

Finally, by equating *f* to the well-known expression for the field enhancement factor in spherical volume [Eq. (12)],

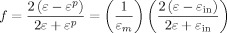
(12)

we find functional form for the effective interior dielectric constant *ε*_in_ [Eq. (13)],

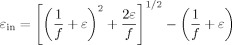
(13)

which may be viewed as dielectric continuum approximation of the solute–solvent system at close proximity, on the order of the chromophore radius. Figure 4 plots the resulting dependence of *ε*_in_ on *ε* for the experimental values of *p* obtained for prodan (filled symbols) and C153 (open symbols). The range of bulk dielectric constant values used in our measurements, *ε*=2.38*–*47.6, lies between the dashed vertical lines, whereas the corresponding “internal” dielectric constant, *ε*_in_=1.5–2.4 agrees well with the commonly assumed value, *ε*_in_=*n*_solute_^2^≈2.[2a,^7^]

**Figure 4:**
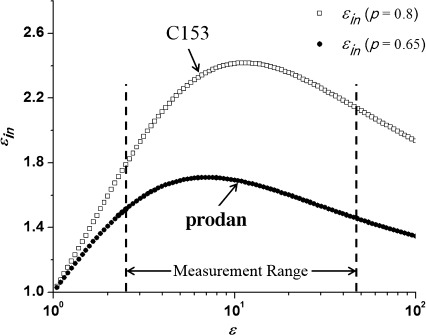
Functional dependence of *ε*_in_ from Equation (13) for experimental values of *p*.

We conclude that this new all-optical method provides an improved quantitative estimation of the average strength and distribution of the dielectric reaction field acting on a dipolar chromophore in different solvents, along with the values of solute dipole moment and polarizability in the ground- and excited electronic states, including the effective molecular radius. The technique is applicable at ambient temperatures, and does not rely on fluorescence emission or externally applied fields, making it a versatile alternative to standard techniques. A simple phenomenological model based on the continuum dielectric solution of Maxwell’s equations is in good quantitative agreement with experimental observations. Finally, our results indicate that the effective dielectric constant near the chromophore exhibits a unique functional dependence on the bulk dielectric constant, which may yield valuable insight into local intermolecular interactions in solvated dipolar systems.

## References

[1] Lakowicz JR (2006). Principles of Fluorescence Spectroscopy.

[2a] Fried SD, Wang LP, Boxer SG, Ren P, Pande VS (2013). J. Phys. Chem. B.

[2b] Onsager L (1936). J. Am. Chem. Soc.

[2c] Vauthey E, Holliday K, Wei C, Renn A, Wild UP (1993). Chem. Phys.

[2d] Renge I (1992). Chem. Phys.

[3a] Samanta A, Fessenden RW (2000). J. Phys. Chem. A.

[3b] Nemkovich NA, Baumann W (2007). J. Photochem. Photobiol. A.

[4a] Saggu M, Levinson NM, Boxer SG (2011). J. Am. Chem. Soc.

[4b] Park ES, Andrews SS, Hu RB, Boxer SG (1999). J. Phys. Chem. B.

[5] Drobizhev M, Makarov NS, Tillo SE, Hughes TE, Rebane A (2011). Nat. Methods.

[6] Rebane A, Drobizhev M, Makarov NS, Beuerman E, Haley JE, Krein DM, Burke AR, Flikkema JL, Cooper TM (2011). J. Phys. Chem. A.

[7] Brunschwig BS, Ehrenson S, Sutin N (1987). J. Phys. Chem.

[8] Gaussian 09, Revision B.01, **2009**

[9] Bosque R, Sales J (2002). J. Chem. Inf. Comput. Sci.

[10] Baumann W, Nagy Z (1993). Pure Appl. Chem.

[11] Chowdhury A, Locknar SA, Premvardhan LL, Peteanu LA (1999). J. Phys. Chem. A.

[12] Moylan CR (1994). J. Phys. Chem.

[13] Balter A, Nowak W, Pawelkiewicz W, Kowalczyk A (1988). Chem. Phys. Lett.

[14] Samanta A, Fessenden RW (2000). J. Phys. Chem. A.

[15a] Horng ML, Gardecki JA, Papazyan A, Maroncelli M (1995). J. Phys. Chem.

[15b] Smirnov SN, Braun CL (1998). Rev. Sci. Instrum.

[15c] Maroncelli M, Fleming GR (1987). J. Chem. Phys.

[16] Kumar PV, Maroncelli M (1995). J. Chem. Phys.

[17] Schmollngruber M, Schroder C, Steinhauser O (2014). Phys. Chem. Chem. Phys.

[18] Vallee RAL, Van Der Auweraer M, De Schryver FC, Beljonne D, Orrit M (2005). ChemPhysChem.

