# Supplementary material for: Two-Photon Voltmeter for Measuring a Molecular Electric Field
Source: Angew Chem Int Ed Engl. 2015 May 8;54(26):7582–6. doi: 10.1002/anie.201502157 (PMC4510705; doi:10.1002/anie.201502157)
Supplement: Supplementary file 1 [file anie0054-7582-sd1.pdf]

Angewandte Chemie
Eine Zeitschrift der Gesellschaft Deutscher Chemiker

Supporting Information

Two-Photon Voltmeter for Measuring a Molecular Electric Field**

Aleksander Rebane, Geoffrey Wicks, Mikhail Drobizhev, Thomas Cooper, Aleksander Trummal, and Merle Uudsemaa*

anie_201502157_sm_miscellaneous_information.pdf

Supplementary Information

Materials and 1-Photon Absorption Measurements

Toluene (> 99.9%), DMSO (> 99.9%), C153 (99%) and PRODAN (98%) were purchased from Sigma-Aldrich and used as received. Linear spectroscopic measurements were performed on a Perkin Elmer Lambda 950 spectrophotometer in 1 cm spectroscopic grade quartz cuvettes for all mixtures of Toluene and DMSO in ratios given in **Table S1**. Extinction coefficients were measured by the weight method.

Table S1: Solvent mixtures used for each chromophore (e.g. 7:1 indicates 7 parts Toluene to 1 part DMSO by volume). X indicates that a 1PA and 2PA spectroscopic measurements were performed in that solvent mixture.

	**Solvent Volume Ratios (Toluene:DMSO)**													
	**8:0 (Toluene)**	**60:1**	**25:1**	**15:1**	**10:1**	**7:1**	**10:2**	**6:2**	**5:3**	**4:4**	**3:5**	**2:6**	**1:7**	**0:8 (DMSO)**
**PRODAN**	X	X	X	X	X	X	X	X	X	X	X	X	X	X
**C153**	X		X	X		X		X	X	X		X		X
**ε**	2.4	3.1	4.1	5.2	6.5	8.0	9.9	14	19	25	31	36	42	48

2-Photon Absorption Measurements

Spectral shapes were obtained using the two-photon excited fluorescence method by employing a tunable optical parametric oscillator (OPO, Insight DeepSee, Spectra Physics) with a step size of 1 nm over the ranges of 680-900 nm (PRODAN) and 700-1025 nm (C153). Fluorescence was collected at a right angle to the incident beam with a PMT. Power dependence was measured at each wavelength with a stepper motor-controlled variable neutral density filter wheel (Thorlabs) that measured the fluorescence intensity for 20 steps over the full range of the wheel (OD 2). The intercept corresponding to the linear fit to the log-log plot of the fluorescence vs. incident power data was used to determine the relative value of the 2PA cross section at each wavelength. Only those points were used where the slope of the power dependence was verified to be in the range 2.00 ± 0.05. Correction for the beam shape was determined by imaging the beam at each wavelength with a CCD camera (Stingray, Basler). Pulse durations were measured using a home-built second harmonic generation autocorrelator using BBO as the doubling material, and were in the range 90-120 fs. The pulse repetition rate of the laser is 80 Mhz, and was chopped down to 70 Hz using a mechanical chopper wheel to alleviate thermal effects in the sample. Excitation power values were adjusted for the solvent absorption based on the linear spectroscopic measurements of each respective mixture. Absolute cross section values were measured for PRODAN (C153) at 750 nm relative to BDPAS (Fluorescein) using the reference method as described in detail earlier[1].

2-Level Model Description

Degenerate two-photon absorption is formally described via second order perturbation theory with the sum-over-states expression[2]

$$\sigma_2^{(i\to f)}\left(\nu/2\right) = \frac{2(2\pi f)^4 \left(\nu/2\right)^2}{(nhc)^2} \left\langle \left| \sum_m \frac{(\vec{\mu}_{im}\bullet\vec{e})(\vec{\mu}_{mj}\bullet\vec{e})}{\left(\nu_{im} - \nu/2\right)} \right|^2 \right\rangle_\Omega g_j(\nu), \tag{S1}$$

where σ_2 is the 2PA cross section, $v/2$ is the two-photon excitation energy, v_{im} is the transition energy between states i and m, h is Planck's constant, c is the speed of light in vacuum, n is the refractive index of the medium, $\vec{e}$ is the polarization unit vector of the optical electric field, $\vec{\mu}_{im}$, $\vec{\mu}_{mj}$ are the dipole moments between states i and m and between states m and j, respectively (note that if $i = m$ or if $m = j$, these are permanent dipole moments), $g_j(\nu)$ is the transition line shape of the final state, f is the local field factor, taken here to be the Lorentz factor, $\frac{n^2+2}{3}$, and $\langle\ \rangle_\Omega$ represents isotropic averaging over all orientations of the absorbing molecules relative to the direction of $\vec{e}$. For the 2-level system approximation, $i = 0$, $j = 1$, and only the indices $m = 0,1$ are included in the summation, so that

$$\sigma_2^{2-state}\left(\nu/2\right) = \frac{2\left(2\pi f\right)^4}{15\left(hcn\right)^2}\left|\vec{\mu}_{10}\right|^2\left|\Delta\vec{\mu}_{10}\right|^2\left(2\cos^2\beta+1\right)g\left(\nu\right), \tag{S2}$$

where $\Delta\vec{\mu}_{10}$ is the permanent dipole moment difference between the ground (*0*) and first excited (*1*) states, and β is the angle between the $\Delta\vec{\mu}_{10}$ and $\vec{\mu}_{10}$ vectors. Note here that the averaging over Ω assumes an isotropic distribution of molecular orientations in solution. Finally, using the relationship between the transition moment and molar extinction coefficient, $\varepsilon_M\left(\nu\right)$ [3],

$$\left|\vec{\mu}_{10}\right|^2 g\left(\nu\right) = \frac{3\cdot 10^3\left(ln10\right)hcn\varepsilon_M\left(\nu\right)}{\left(2\pi\right)^3 N_A\nu f^2}, \tag{S3}$$

where N_A is Avogadro's number, and assuming that $\beta \approx 0$, the 2-level 2PA cross section is given as

$$\sigma_2^{2-state}\left(\nu/2\right) = \left|\Delta\vec{\mu}_{10}\right|^2\varepsilon_M\left(\nu\right)\left(\frac{12\pi\cdot 10^3\left(ln10\right)f^2}{5hcn\nu N_A}\right). \tag{S4}$$

Rearranging for $\Delta\vec{\mu}_{10}$ and converting into the units of Eqn. (1) in the main paper,

$$\left|\Delta\vec{\mu}_{10}\right| = \left(\frac{5hc^2N_A\cdot 10^{-50}}{12\pi\cdot 10^3\cdot 10^{-7}\left(ln10\right)}\right)^{1/2}\frac{10^{18}}{f}\sqrt{\frac{n\sigma_2^{2-state}\left(2\lambda\right)}{\lambda\varepsilon_M\left(\lambda\right)}} = \left(\frac{4548}{f}\right)\sqrt{\frac{n\sigma_2^{2-state}\left(2\lambda\right)}{\lambda\varepsilon_M\left(\lambda\right)}}. \tag{S5}$$

In Eqn. (S5), the factors 10^{-50}, 10^{18}, 10^3, and 10^{-7} correspond respectively to the unit conversions of σ_2 ($cm^4\cdot s$→GM), $\Delta\vec{\mu}_{10}$ (StatC·cm→D), ε_M ($cm^2\cdot mol^{-1}$→ $L\cdot mol^{-1}\cdot cm^{-1}$), and λ (cm→nm).

MATLAB Optimization Routine Description

The model line shape is calculated based on Eq. (10) from the main text, where the peak transition energy and width are extracted from the fit to Eq. (7) for approximately 250 million combinations of the 5 unknown parameters over a broad input value interval (see below). The program then subtracts these model results from the experimental data to calculate the residual, and stores the product of the absolute value of the residuals (peak and width) in an array corresponding to the parameter values used for that particular calculation. The parameter interval ranges are then iteratively reduced until convergence of the results is achieved within the expected experimental uncertainty (±10%). The coordinates of the minimum value in

this final 5-D array then correspond to the parameter values that best reproduce the experimental spectra, according to our model. Note that the experimental data are sufficient to solve simultaneously for 5 parameters, given that for convergence, a global fit must be obtained for the values of the peak transition energy and its first 2 derivatives vs. ε, as well as the values for the width and its first derivative vs. ε (see **Figures S4, S5**). The initial parameter variation intervals for both chromophores were the same and are given as follows:

$$0.05 \le p \le 0.95,\ 3\ D \le \mu_{vac}\left(S_0\right) \le 9\ D,\ 10\ \text{Å}^3 \le \alpha_0\left(S_0\right) \le 60\ \text{Å}^3,\ 3\ \text{Å} \le a_0 \le 10\ \text{Å},\ 0.01\ \text{Å} \le \Delta a \le 3\ \text{Å}$$

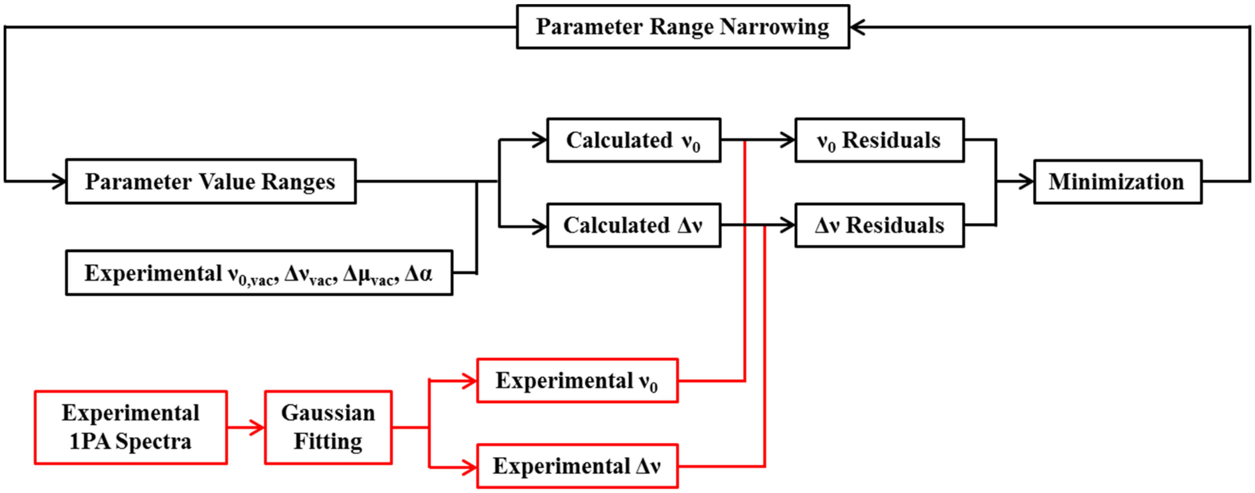

Figure S1: Flow chart outlining MATLAB parameter optimization procedure.

Computational Details

Ground state conformational analysis of Prodan and Coumarin C153 compounds in a gas phase has been carried out at BP86/TZVPP level using the Turbomole[4] software package. The resulting structures were re-optimized at B3LYP/6-311G(d,p) level in Gaussian09[5] and the absence of imaginary frequencies was confirmed. Vertical excited state properties of the lowest singlet states were obtained from Gaussian09 TDDFT calculations using the Coulomb Attenuated Method CAM-B3LYP functional and the 6-311++G(d,p) basis set for the ground state geometries of Prodan and Coumarin 153. For Prodan, adiabatic properties of the first excited singlet state were also explored by optimizing the structure of the excited state at TD/CAM-B3LYP/6-311++G(d,p) level. The effects of solvation on excited state properties of Prodan were studied in toluene and dimethyl sulfoxide (DMSO) using linear response (LR) equilibrium IEF-PCM solvation (toluene and DMSO), as well as non-equilibrium LR and state-specific IEF-PCM solvation (DMSO) combined with the TDDFT approach. Excited-state dipole moments were obtained from relaxed excited-state electron densities computed by Z-vector method as implemented in Gaussian09. Excited-state polarizability was computed as the numerical derivative of the dipole moment. The isotropic average of the polarizability in the ground and excited states is used for comparison in Table 1. Unless specified otherwise, the results correspond to the lowest energy conformers of Prodan and Coumarin 153 (ground state gas-phase reference).

Results, Fit Analysis and Extrapolation

Figure S1 shows the measured 1PA (solid line) and 2PA (black markers) spectra for PRODAN (left) and C153 (right), in all measured solvent mixtures. C153 was measured in fewer mixtures for the sake of expediency, once it was determined that this was sufficient for reasonable determination of the parameters.

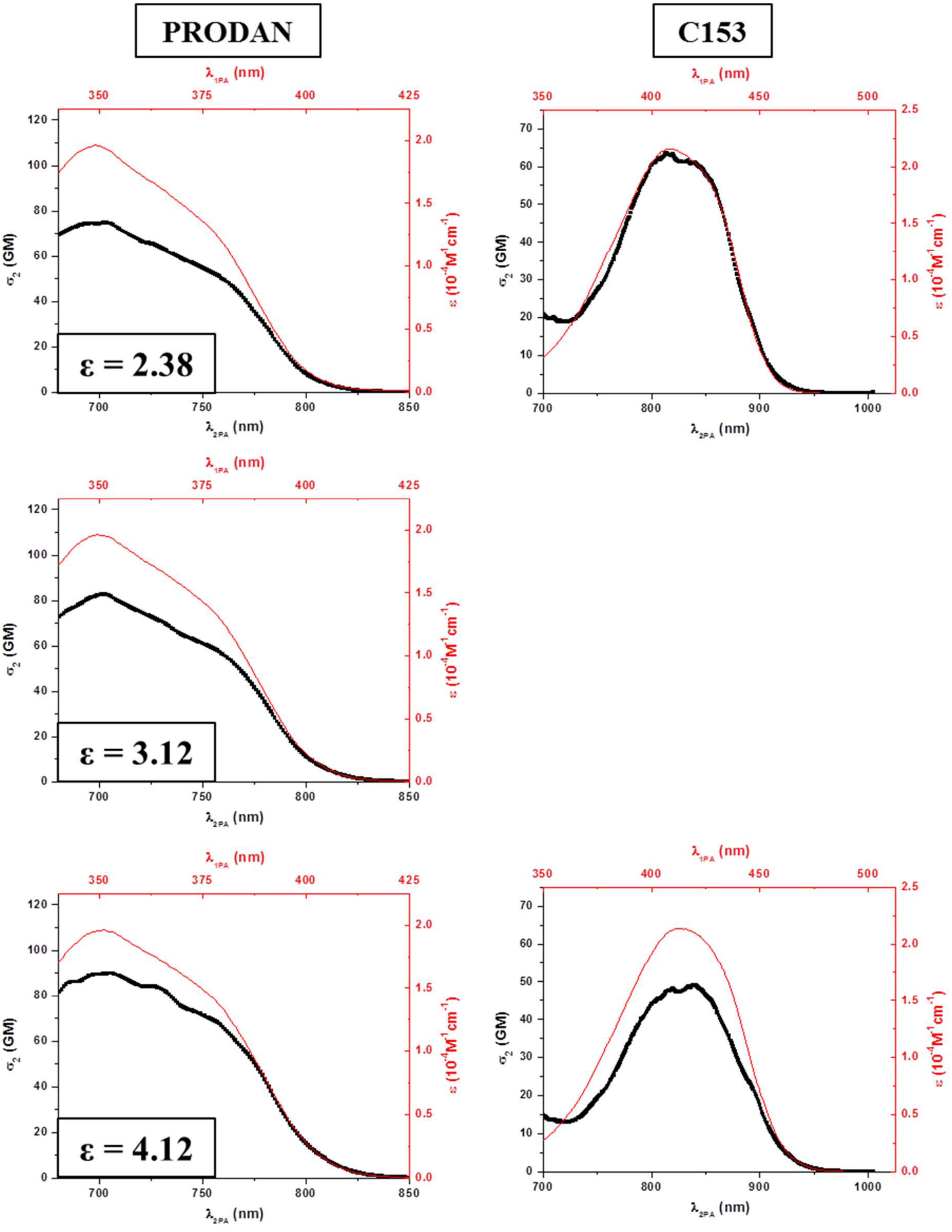

PRODAN
C153
λ1PA (nm)
λ2PA (nm)
σ2 (GM)
ε (10-4M-1cm-1)
ε = 2.38
ε = 3.12
ε = 4.12

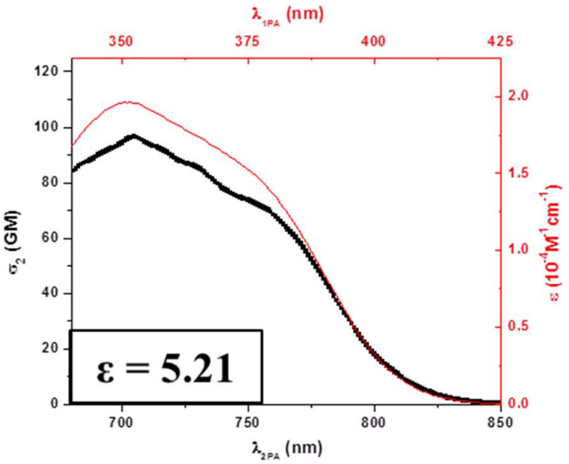
λ1PA (nm)
350
375
400
425
σ2 (GM)
ε (10^-4 M^-1 cm^-1)
ε = 5.21
λ2PA (nm)
700
750
800
850

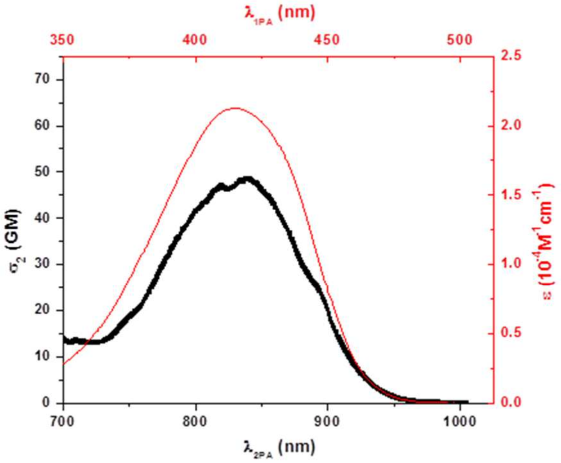
λ1PA (nm)
350
400
450
500
σ2 (GM)
ε (10^-4 M^-1 cm^-1)
λ2PA (nm)
700
800
900
1000

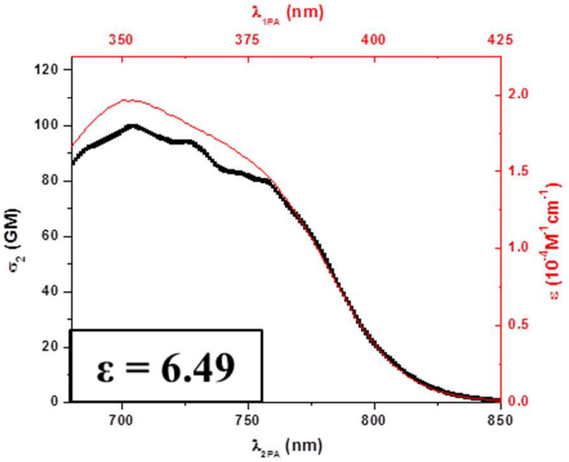
λ1PA (nm)
350
375
400
425
σ2 (GM)
ε (10^-4 M^-1 cm^-1)
ε = 6.49
λ2PA (nm)
700
750
800
850

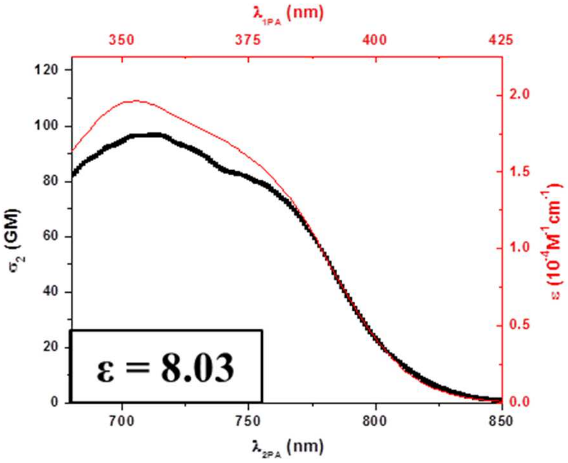
λ1PA (nm)
350
375
400
425
σ2 (GM)
ε (10^-4 M^-1 cm^-1)
ε = 8.03
λ2PA (nm)
700
750
800
850

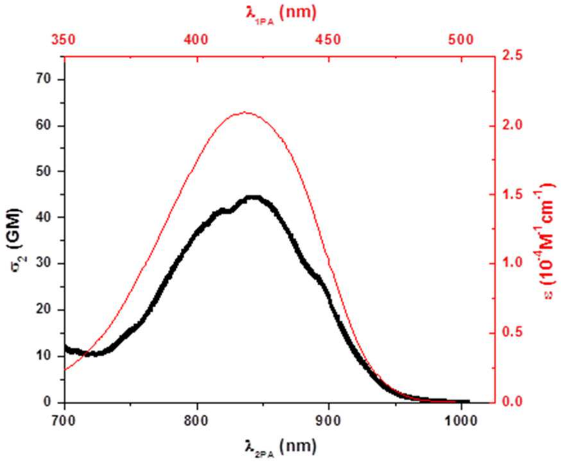
λ1PA (nm)
350
400
450
500
σ2 (GM)
ε (10^-4 M^-1 cm^-1)
λ2PA (nm)
700
800
900
1000

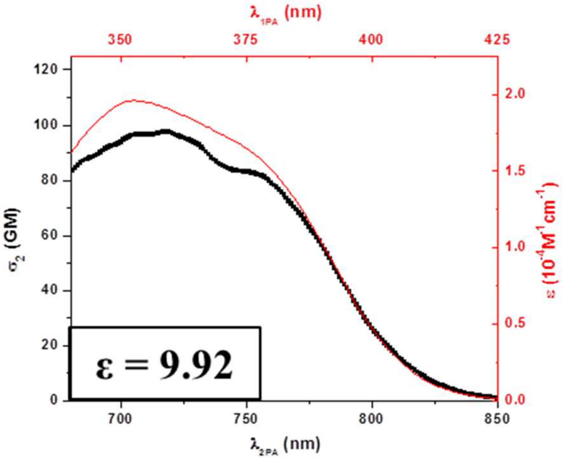
λ_{1PA} (nm)
350 375 400 425
σ_2 (GM)
0 20 40 60 80 100 120
ε ($10^{-4}M^{-1}cm^{-1}$)
0.0 0.5 1.0 1.5 2.0
ε = 9.92
700 750 800 850
λ_{2PA} (nm)

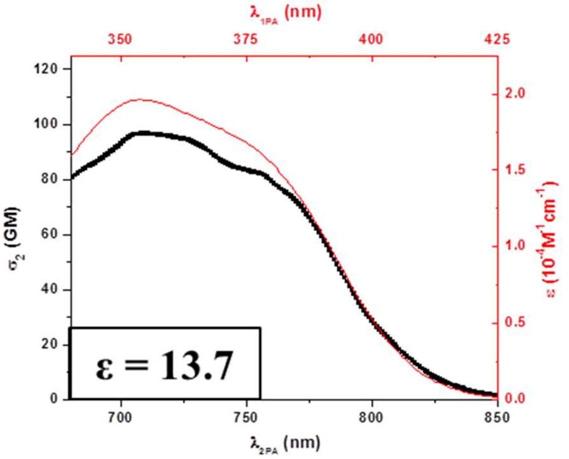
λ_{1PA} (nm)
350 375 400 425
σ_2 (GM)
0 20 40 60 80 100 120
ε ($10^{-4}M^{-1}cm^{-1}$)
0.0 0.5 1.0 1.5 2.0
ε = 13.7
700 750 800 850
λ_{2PA} (nm)

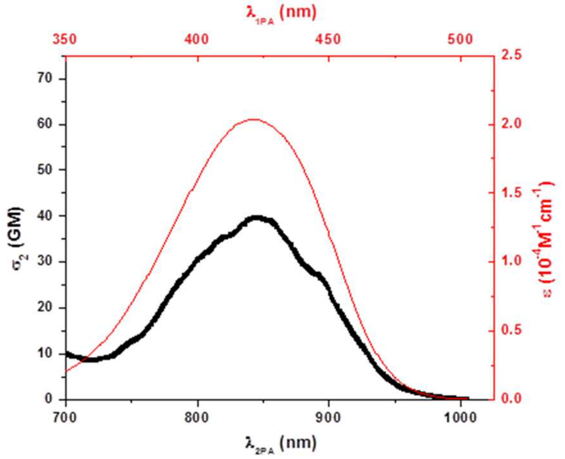
λ_{1PA} (nm)
350 400 450 500
σ_2 (GM)
0 10 20 30 40 50 60 70
ε ($10^{-4}M^{-1}cm^{-1}$)
0.0 0.5 1.0 1.5 2.0 2.5
700 800 900 1000
λ_{2PA} (nm)

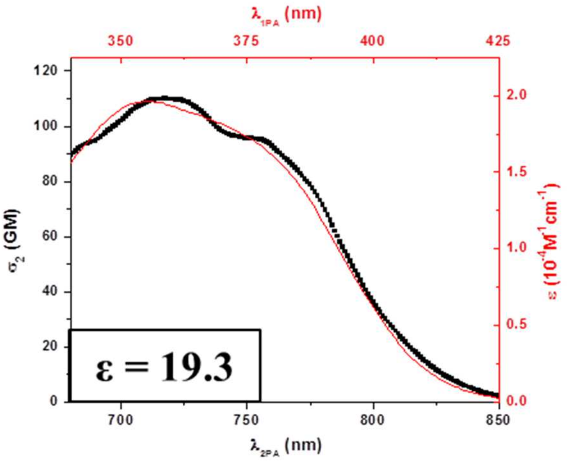
λ_{1PA} (nm)
350 375 400 425
σ_2 (GM)
0 20 40 60 80 100 120
ε ($10^{-4}M^{-1}cm^{-1}$)
0.0 0.5 1.0 1.5 2.0
ε = 19.3
700 750 800 850
λ_{2PA} (nm)

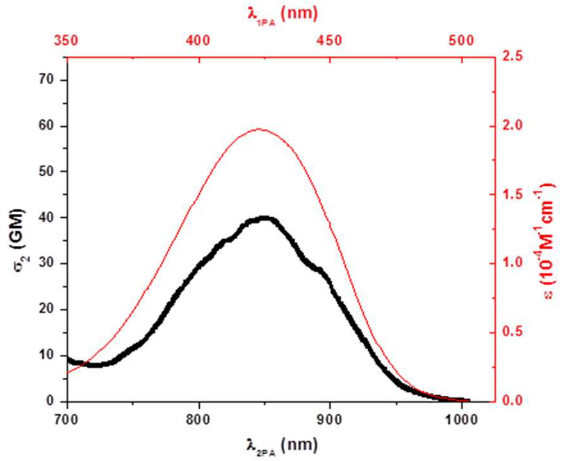
λ_{1PA} (nm)
350 400 450 500
σ_2 (GM)
0 10 20 30 40 50 60 70
ε ($10^{-4}M^{-1}cm^{-1}$)
0.0 0.5 1.0 1.5 2.0 2.5
700 800 900 1000
λ_{2PA} (nm)

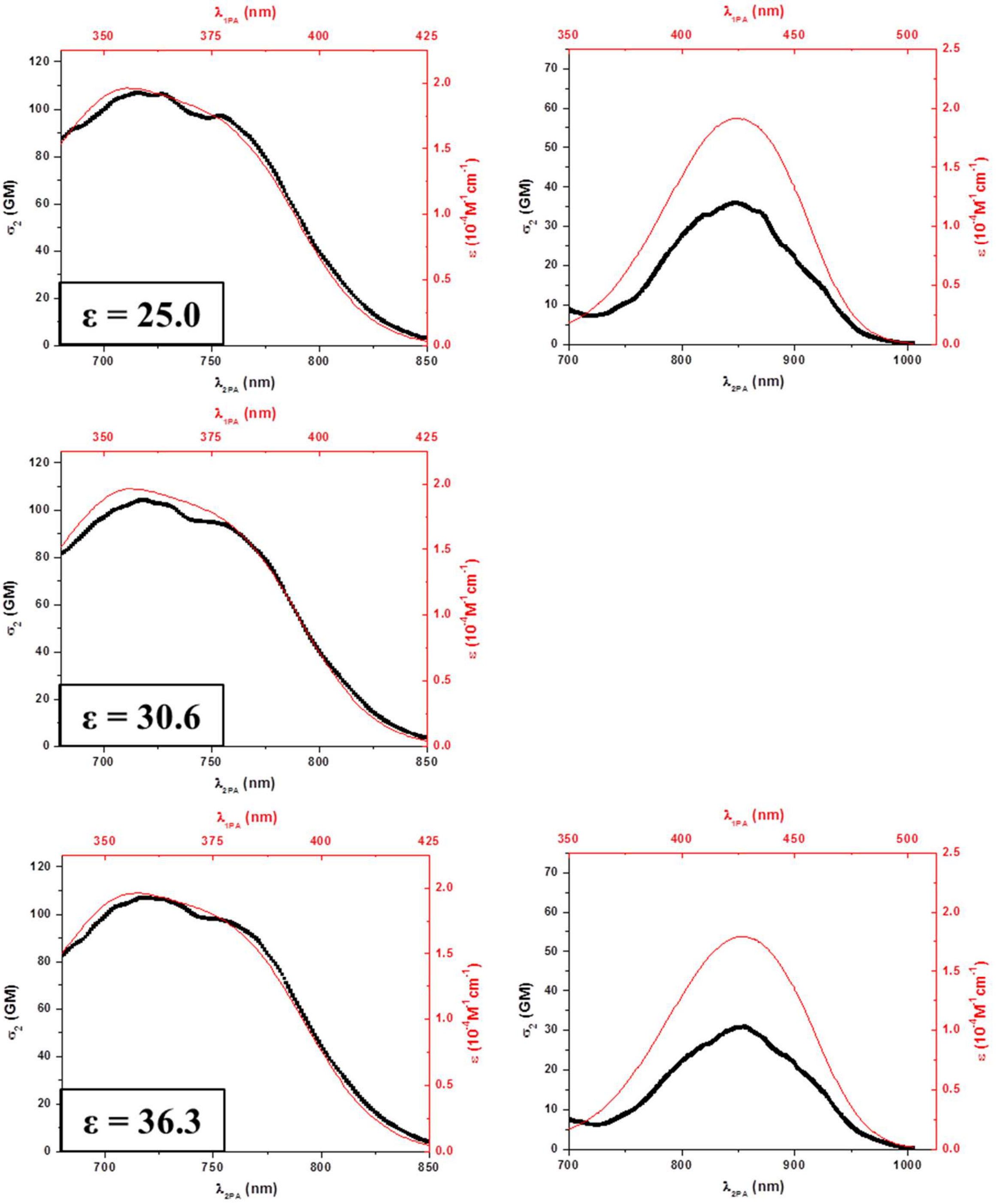
ε = 25.0
ε = 30.6
ε = 36.3
σ_2 (GM)
λ_{2PA} (nm)
λ_{1PA} (nm)
ε ($10^{-4}M^{-1}cm^{-1}$)

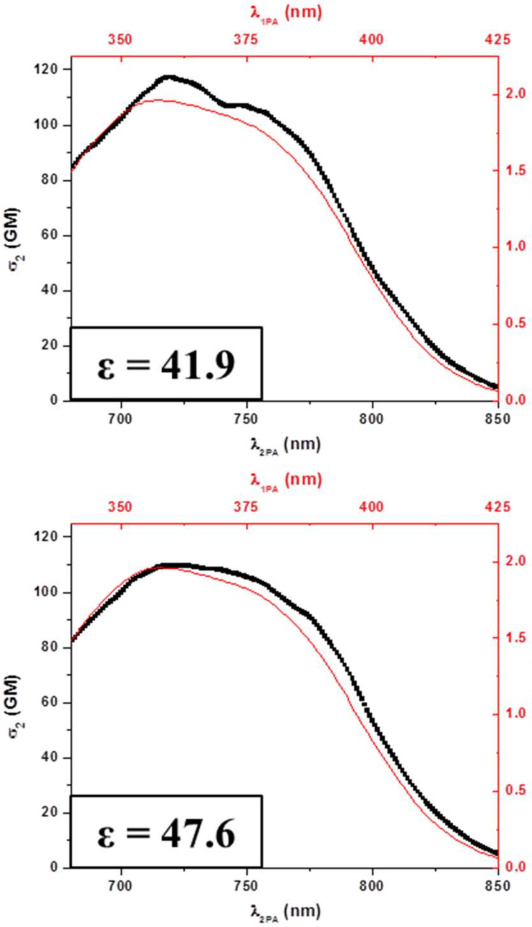

Figure S2: Measured 1PA (solid line) and 2PA (black markers) spectra, respectively, for PRODAN (left) and C153 (right). Dielectric constant of the solvent mixture is indicated in the text box in the left column.

Figure S3 compares the 2PA between solvent mixtures for PRODAN (left) and C153 (right).

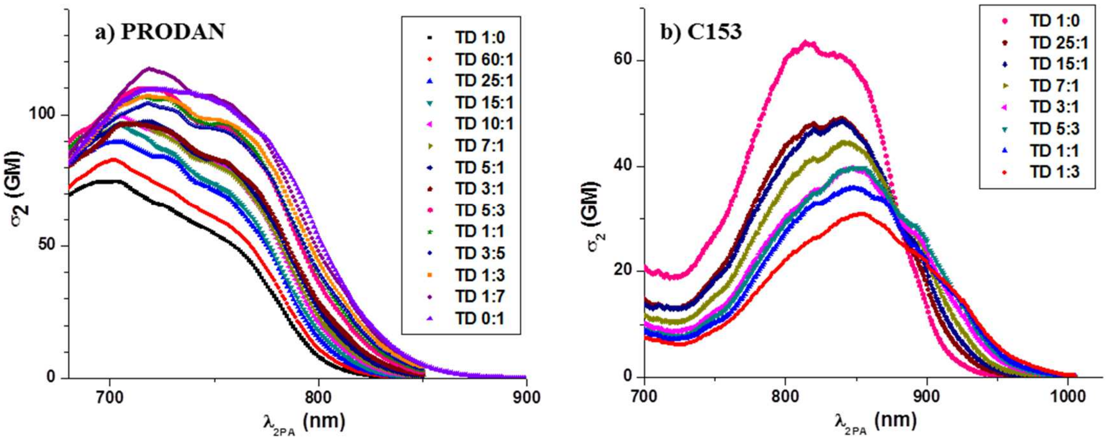

Figure S3: 2PA spectra for a) PRODAN and b) C153. The legend indicates the solvent mixture used, e.g. TD 7:1 is 7 parts Toluene to 1 part DMSO by volume.

Figures S4 and S5 show the dependence of the 1PA peak energy (Gaussian fit for the lowest energy transition) on the dielectric constant for PRODAN (left) and C153 (right). The symbols are experimental data and the red curves show the fits to the data.

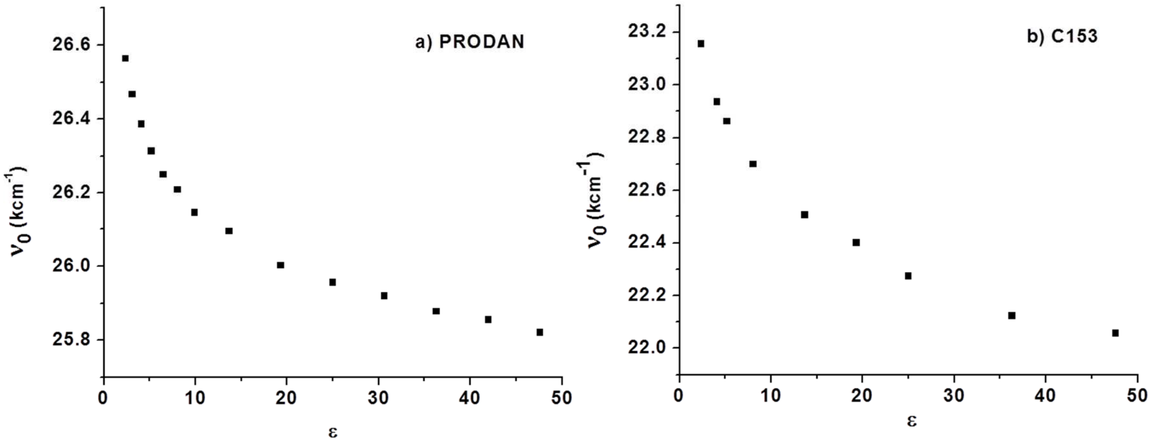

Figure S4: Peak transition energies from the Gaussian fit to experimental 1PA spectra for different solvent mixtures; a) PRODAN, b) C153.

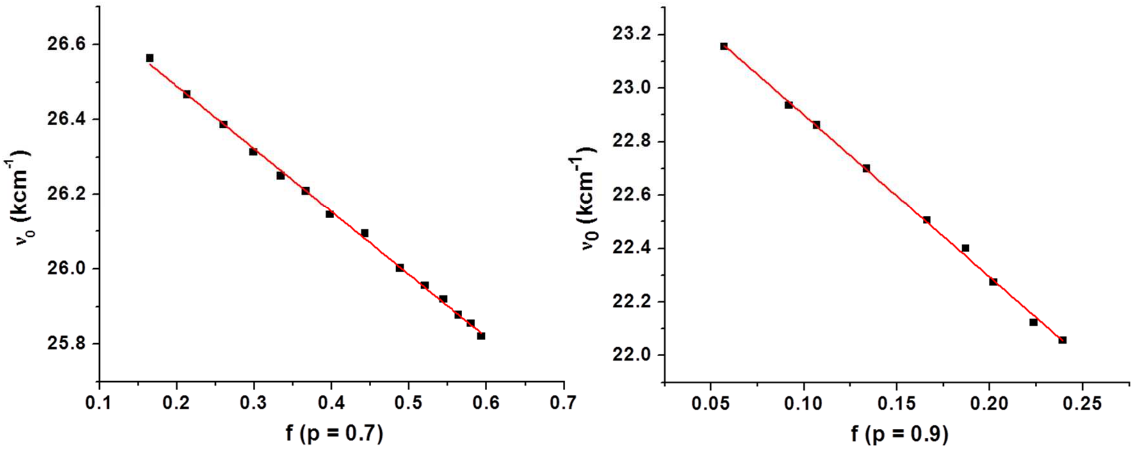

Figure S5: Peak transition energies from the Gaussian fit to experimental 1PA spectra for different solvent mixtures, plotted against the linearizing function f(p) defined in Eqn. (5) of the main paper; a) PRODAN, b) C153. Red lines indicated fits used for extraction of ν_{vac}.

Figure S6 shows the dependence of the 1PA lowest energy band width (Gaussian fit for the lowest energy transition) on the dielectric constant for PRODAN (left) and C153 (right). The symbols are experimental data and the red curves show the fits to the data.

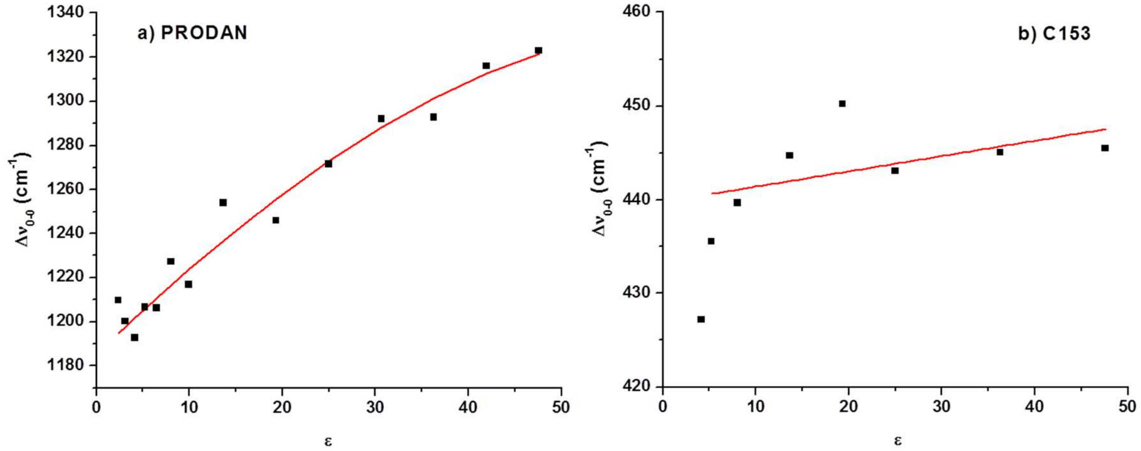

Figure S6: Widths of lowest energy band from the Gaussian fits to the experimental 1PA spectra for different solvent mixtures; a) PRODAN, b) C153. Red curves indicate fits used to extract $\Delta\nu_{vac}$.

Figure S7 shows the final predicted 1PA spectra (black markers) and fits to the experimental spectra (red lines). The model predictions almost exactly reproduce the experimental data. This is to be expected, as we optimized the parameters in order to reproduce the 1PA spectral data in the MATLAB routine.

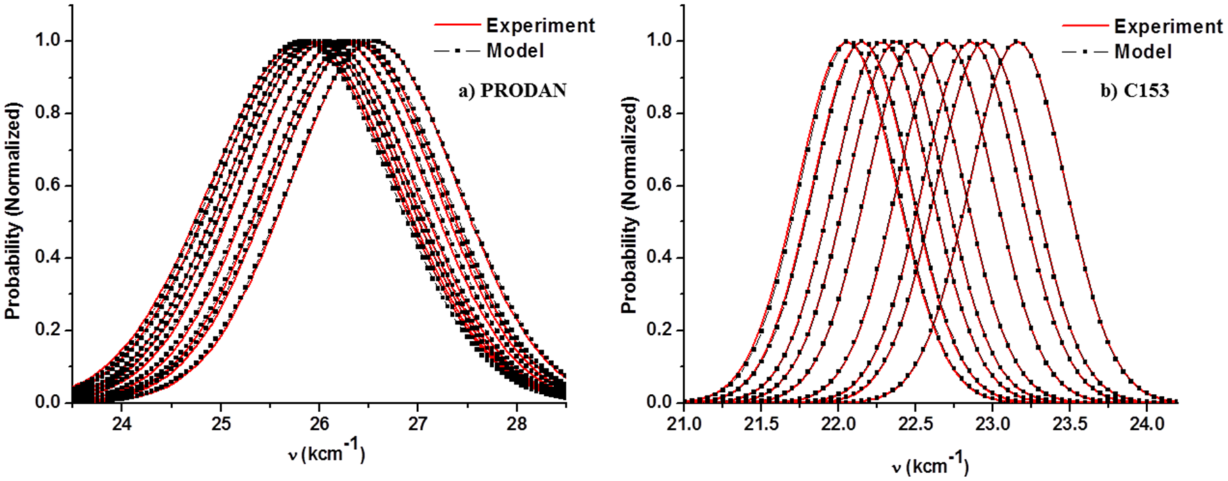

Figure S7: Lowest 1PA transition band shapes, experiment (red) compared with model prediction (black) for a) PRODAN (only the mixtures that coincide with the C153 measurements are shown for clarity) and b) C153.

Discussion of Cavity Model:

The empirical formula for the power dependence of the interior dielectric constant on that of the bulk solvent was chosen in order to account for close-range intramolecular interactions while still maintaining the conditions defining the reaction field. These conditions maintain that: 1) $\varepsilon_{in} \leq \varepsilon$ for all dielectric

constants so that the reaction field points in the direction of $\vec{\mu}_0$ and therefore lowers, instead of increasing, the energy of the system, and; 2) $\lim_{\varepsilon \to 1} E_{reac} = 0$, so that in the vacuum limit the reaction field goes to zero. It is commonly assumed that the interior of the cavity is vacuum, but this does not account for the effect of close-range interactions which can sometimes play a dominant role. We take the approach of assuming that the "dielectric" response of the solute molecule is related to the dielectric properties of the solvent. How the value for *p* is related to the type and magnitude of close-range interaction remains to be determined. However, an interesting comparison can be made between our power dependence and the Lippert-Mataga orientational polarizability function *Δf*, given in Eq. (S6), where *n* is the solvent refractive index, taken here to be the average value of 1.488[6]. **Figure S8** compares the ε dependence of *f* from Eq. (5) for PRODAN (*p* = 0.65) and C153 (*p* = 0.8), and the Lippert-Mataga *Δf*.

$$\Delta f = \frac{2(\varepsilon - 1)}{2\varepsilon + 1} - \frac{2(n^2 - 1)}{2n^2 + 1} \quad \text{(S6)}$$

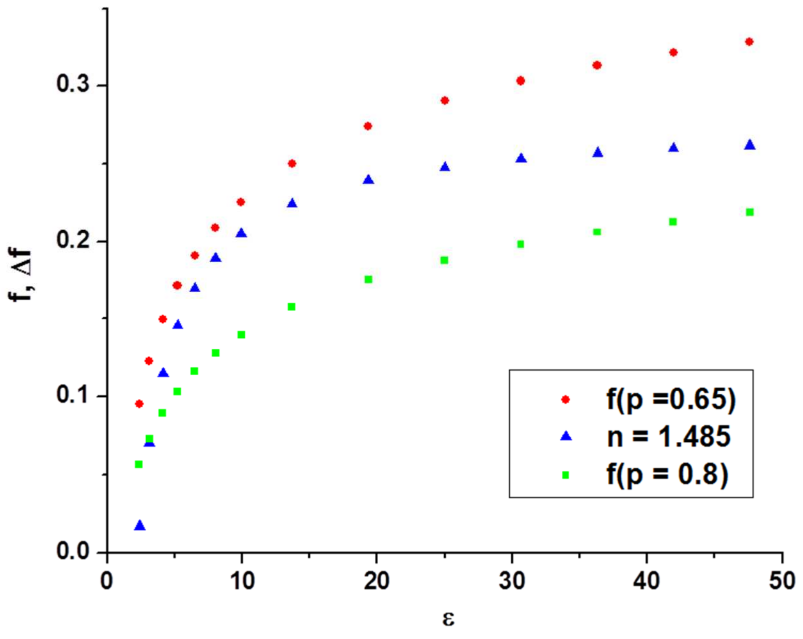

Figure S8: Comparison between Lippert-Mataga *Δf* (blue triangles) and *f(p)* empirical solvent description for PRODAN (p = 0.65, red circles) and C153 (p = 0.8, green squares).

It can be observed that the *Δf* function provides a reasonable estimate to the behavior, but saturates more rapidly than our function *f* as a consequence of the constant (vacuum) values for the interior dielectric

constant and refractive index. Changing the value of p slightly from 0.65 to 0.8 yields a lower scaling for the reaction field, perhaps indicative of a different electrostatic response to the solvent environment.

Uncertainty Estimation:

1) Experimentally determined parameters (ν_{vac}, $\Delta\nu_{vac}$, $\Delta\mu_{vac}$, $\Delta\alpha$):

The error in ν_{vac} (**Figure S5**) is expected to be primarily determined by the accuracy of the Gaussian fitting. This is because: 1) f(p) was optimized in order to minimize the standard error between the linear fits to the peak transition energy dependence (8 cm^{-1} and 15 cm^{-1} for PRODAN and C153, respectively); and 2) The lack of resolution of the peaks in the absorption band prevented locating the peaks with an accuracy less than these standard errors. The uncertainty in ν_{vac} is therefore estimated at ~5% of the width of the total 1PA band, or ±200 cm^{-1}.

The main contribution to the uncertainty in the accuracy of our optimized parameter values is due to that associated with the reference standards used for the determination of 2PA cross section values. This uncertainty is estimated at ±10% of σ_2. This corresponds to approximately ±5% of $\Delta\mu$, and is a systematic error expected to propagate to all solvent mixtures. Therefore, the result of this error is a systematic discrepancy in $\Delta\mu_{vac}$ of ±5%.

2) Parameters determined through global 1PA fitting:

References

[1] N. S. Makarov, M. Drobizhev, A. Rebane, *Optics Express* **2008**, *16*, 4029-4047.
[2] B. J. Orr, J. F. Ward, *Mol. Phys.* **1971**, *20*, 513-526.
[3] D. Toptygin, *Journal of Fluorescence* **2003**, *13*, 19.
[4] TURBOMOLE V6.4 2012, a development of University of Karlsruhe and Forschungszentrum Karlsruhe GmbH, 1989-2007, TURBOMOLE GmbH, since 2007; available from http://www.turbomole.com,
[5] Gaussian 09, Revision B.01, **2009**, M. J. Frisch, et al., Gaussian, Inc., Wallingford, CT
[6] J. R. Lakowicz, *Principles of Fluorescence Spectroscopy, Third Edition*, Springer, New York, NY, **2006** (and references therein).
